# Presacral Myelolipoma Mimicking Liposarcoma Complicated by Massive Intraoperative Hemorrhage: A Case Report

**DOI:** 10.70352/scrj.cr.26-0452

**Published:** 2026-11-03

**Authors:** Daichi Hanaoka, Shingo Shimada, Yoichi Miyaoka, Kyoko Hanaoka, Tomoya Saito, Yoshifumi Iwaguchi, Shin Ichihara, Ryoji Yokoyama

**Affiliations:** 1 Department of Surgery, Abashiri-Kosei General Hospital, Abashiri, Hokkaido, Japan; 2 Department of Surgery, Otaru General Hospital, Otaru, Hokkaido, Japan; 3 Department of Surgical Pathology, Sapporo-Kosei General Hospital, Sapporo, Hokkaido, Japan; 4 Department of Diagnostic Pathology, Asahikawa Medical University Hospital, Asahikawa, Hokkaido, Japan

**Keywords:** myelolipoma, presacral tumor, liposarcoma, massive intraoperative hemorrhage, internal iliac vein, presacral venous plexus

## Abstract

**INTRODUCTION:**

Myelolipoma is a benign tumor composed of mature adipose tissue and hematopoietic elements. Extra-adrenal lesions can occur in the presacral region and may mimic liposarcoma. Resection of a large presacral tumor can also cause life-threatening hemorrhage. We report a presacral myelolipoma that was difficult to diagnose preoperatively and was complicated by massive intraoperative hemorrhage.

**CASE PRESENTATION:**

A 71-year-old woman had an incidentally detected 75 × 75 × 125-mm presacral mass with mixed fatty and solid components on contrast-enhanced CT. MRI showed restricted diffusion, and fluorodeoxyglucose PET/CT showed mild uptake (maximum standardized uptake value, 3.93). No growth occurred over approximately 3 months. Because liposarcoma could not be excluded, the patient chose open resection without biopsy for diagnosis and treatment. During cranial dissection along the anterior sacrum, massive bleeding occurred from the right internal iliac venous system, and systolic blood pressure decreased to 50–60 mmHg. Manual compression was insufficient, and massive transfusion was initiated. Intraoperatively, a vein communicating with the right internal iliac vein was found to enter the tumor, and the main trunk of the right internal iliac vein was ligated near its junction with the right common iliac vein. Persistent bleeding required ligation of a tumor-entering branch of the right internal iliac artery; the arterial main trunk was preserved. Hemostasis was prioritized, and the tumor was removed in 2 pieces; the capsule was disrupted without macroscopic tumor spillage. Diffuse presacral venous plexus bleeding was controlled with electrocautery and oxidized cellulose. Operative time was 3 h 21 min, and blood loss was 4850 mL; 10 units of leukocyte-reduced red blood cells and 6 units of fresh frozen plasma were transfused. Histopathological examination established the diagnosis of myelolipoma. No vascular complications occurred, and contrast-enhanced CT at approximately 1 year showed no residual or recurrent tumor.

**CONCLUSIONS:**

Presacral myelolipoma should be considered in the differential diagnosis of fat-containing presacral tumors. In large tumors, the relationship with the internal iliac vessels and presacral venous plexus should be carefully assessed, and adequate blood products, early vascular control, and source-specific hemostatic techniques should be prepared before resection.

## Abbreviations

ADC
: apparent diffusion coefficient
BMI
: body mass index
CA19-9
: carbohydrate antigen 19-9
CA125
: cancer antigen 125
CEA
: carcinoembryonic antigen
FDG
: fluorodeoxyglucose
FFP-LR
: leukocyte-reduced fresh frozen plasma
RBC-LR
: leukocyte-reduced red blood cells
SUVmax
: maximum standardized uptake value

## INTRODUCTION

Myelolipoma is a benign tumor composed of mature adipose tissue and hematopoietic elements and most commonly arises in the adrenal gland.^1)^ Adrenal myelolipoma has an estimated autopsy prevalence of approximately 0.08%–0.2% and accounts for 6%–16% of adrenal incidentalomas.^1)^ Extra-adrenal myelolipoma accounts for only approximately 15% of all myelolipomas; however, the presacral or retrorectal space is one of the relatively common sites of extra-adrenal disease.^2–4)^ The presacral space can harbor a wide variety of lesions, including developmental cysts, neurogenic tumors, osseous tumors, teratomas, and soft-tissue tumors such as liposarcoma, making preoperative diagnosis difficult.^4)^

On imaging, presacral myelolipoma often contains an admixture of macroscopic fat and soft-tissue components, and its internal architecture may overlap with that of well-differentiated or dedifferentiated liposarcoma. Furthermore, FDG uptake or diffusion restriction can increase concern for malignancy, although these findings may reflect hematopoietic elements within a benign myelolipoma.^5,6)^ We report a large presacral myelolipoma that was difficult to distinguish from liposarcoma preoperatively and caused massive bleeding from the right internal iliac venous system and presacral venous plexus during resection, with a focus on diagnostic decision-making and hemorrhage control in deep pelvic surgery.

## CASE PRESENTATION

A 71-year-old woman was referred to our department because a pelvic mass was incidentally detected on CT performed during follow-up for cholelithiasis and a gallbladder polyp at a previous hospital. Contrast-enhanced CT and MRI performed by the gynecology department suggested that the lesion was unlikely to originate from the gynecological organs, and she was referred for surgical evaluation.

Her medical history included hypertension, dyslipidemia, atrophic gastritis, cholelithiasis, and a gallbladder polyp. She had no abdominal symptoms. On physical examination, her height was 155.7 cm, body weight was 78.7 kg, and BMI was 32.4 kg/m^2^. Her body temperature was 36.6°C, blood pressure was 137/91 mmHg, and pulse rate was 70 beats/min. The abdomen was soft and non-distended without tenderness, and no palpable mass was detected.

Laboratory examinations showed a white blood cell count of 7500/µL and a C-reactive protein level of 0.23 mg/dL, indicating no significant inflammatory response. There was no anemia or other blood cell count abnormality, and the patient had no history of a hematologic disorder, including hemolytic anemia or a myeloproliferative neoplasm. Imaging showed no hepatosplenomegaly. Tumor marker levels were within normal limits: CEA, 2.7 ng/mL; CA19-9, 2.0 U/mL; and CA125, 13.7 U/mL. Colonoscopy revealed no abnormal findings.

Contrast-enhanced pelvic CT demonstrated a well-defined, lobulated mass measuring 75 × 75 × 125 mm anterior to the sacrum and posterior to the rectum. The mass contained both fatty and solid components (**Fig. 1A** and **1B**). After surgery, retrospective review of the preoperative contrast-enhanced CT revealed a venous branch arising from the right internal iliac vein and coursing toward the tumor (**Fig. 1C**). This radiologic finding could not be definitively correlated with the vessels identified and ligated intraoperatively; the preoperative CT did not establish the precise injured vein or identify the arterial branch. No definite vascular encasement or invasion had been recognized preoperatively. However, the presence or absence of vascular compression, displacement, or adherence could not be determined reliably because the detailed relationship between the tumor and the internal iliac vessels was not prospectively assessed. The venous branch was not recognized as a bleeding risk, and 3D CT vascular reconstruction was not performed; consequently, the risk of massive hemorrhage was not fully anticipated. Pelvic MRI showed heterogeneous high signal intensity on T1- and T2-weighted images, with suppression of the fatty component on fat-suppressed sequences. The solid component showed high signal intensity on diffusion-weighted imaging and low signal intensity on the ADC map, indicating restricted diffusion (**Fig. 2**). FDG PET/CT showed mild abnormal uptake corresponding to the tumor, with a SUVmax of 3.93. No abnormal uptake was observed elsewhere (**Fig. 3**). During approximately 3 months of imaging follow-up after detection, no clear change in tumor size or internal characteristics was observed.

**Fig. 1:**
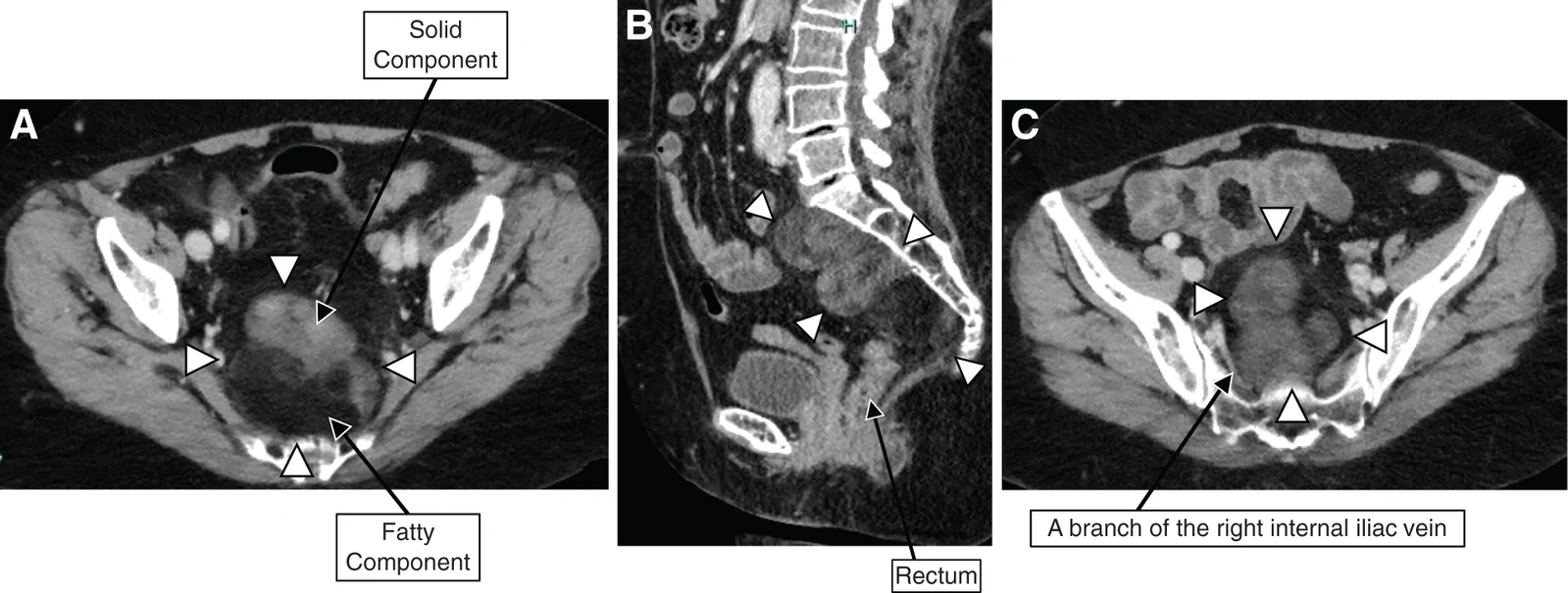
Preoperative CT findings. Arrowheads indicate the tumor. (**A**) Axial view. (**B**) Sagittal view. Contrast-enhanced CT shows a well-defined, lobulated mass in the presacral space containing both fatty and solid components. (**C**) Retrospective review of preoperative contrast-enhanced CT shows a venous branch arising from the right internal iliac vein and coursing toward the tumor; this vessel was not confirmed to be the source of intraoperative bleeding.

**Fig. 2:**
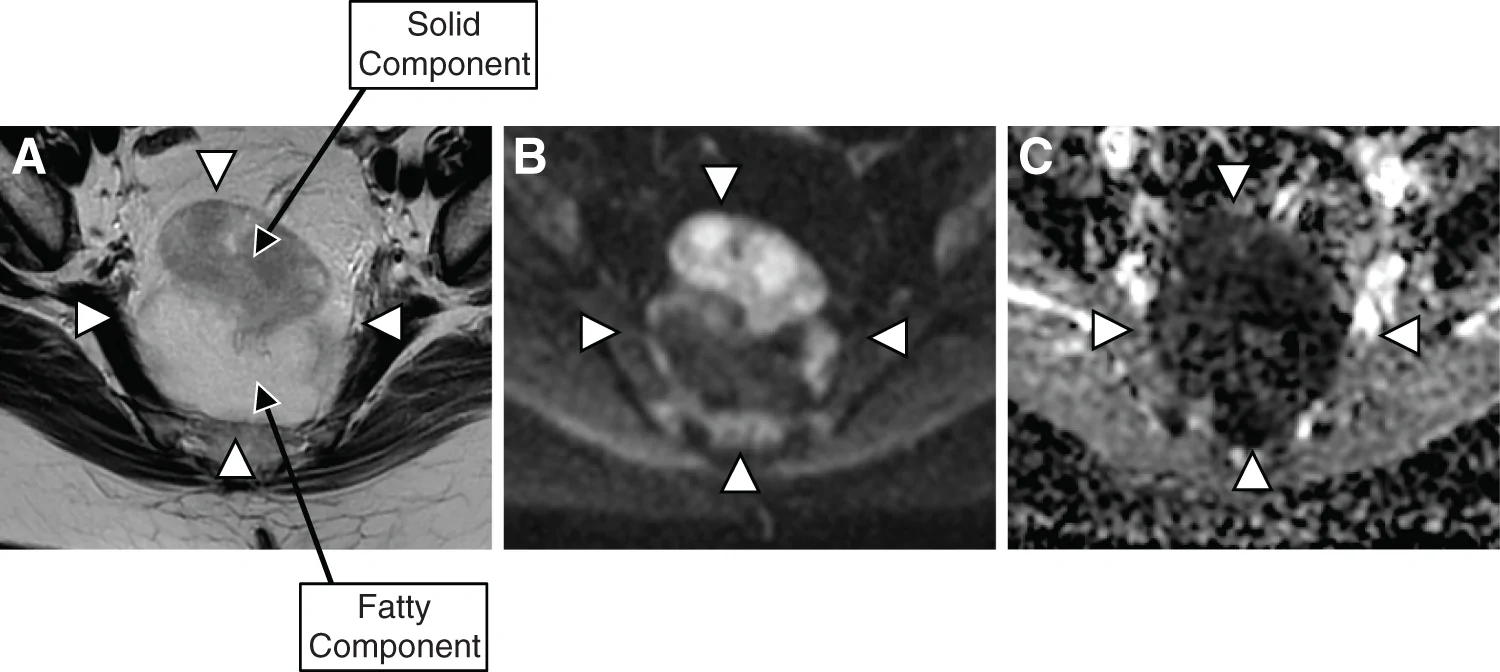
MRI findings. Arrowheads indicate the tumor. (**A**) T2-weighted imaging shows a heterogeneous high-intensity mass. (**B**) Diffusion-weighted imaging and (**C**) ADC mapping demonstrate restricted diffusion within the solid component. ADC, apparent diffusion coefficient

**Fig. 3:**
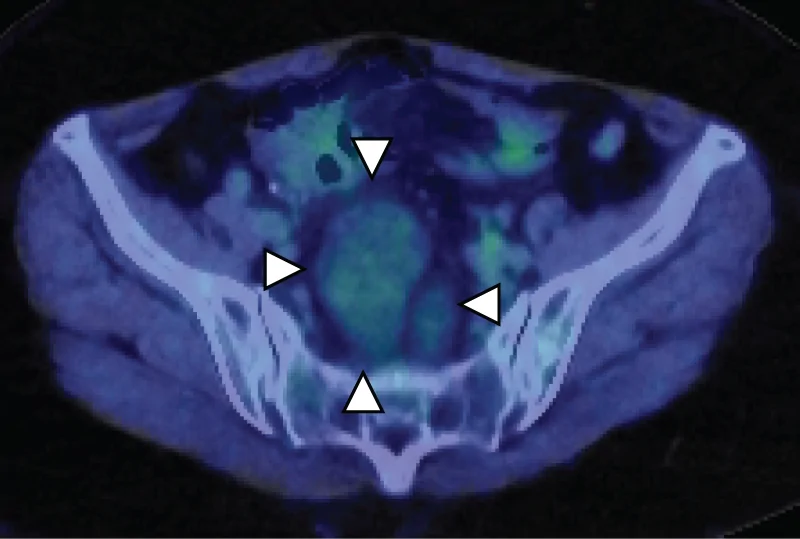
FDG PET/CT findings. The tumor shows mild FDG uptake, with a SUVmax of 3.93. Arrowheads indicate the tumor. FDG, fluorodeoxyglucose; SUVmax, maximum standardized uptake value

The mixed fatty and solid components, diffusion restriction, FDG uptake, and large tumor size made it difficult to exclude liposarcoma. Although the patient was asymptomatic and no short-term growth had been observed, the possibility of malignancy and future defecatory dysfunction due to rectal compression were explained. She preferred definitive diagnosis and resection rather than continued observation. Because resection was selected for both diagnosis and treatment in accordance with her preference, the feasibility of image-guided biopsy was not formally assessed. Owing to the tumor size, deep pelvic location, proximity to the pelvic vessels, and possibility of malignancy, an open approach was selected rather than laparoscopic surgery. A type and screen was performed preoperatively; however, crossmatched RBC-LR units were not reserved, and FFP-LR was to be supplied from routine hospital stock. Because massive pelvic hemorrhage was not anticipated, no pre-emptive proximal vascular control was planned before tumor mobilization.

### Operative findings

Open laparotomy was performed via a lower midline incision. The tumor was located in the retroperitoneal space caudal to the bifurcation of the common iliac arteries and occupied the presacral region. After mobilization of the rectal mesentery, tumor dissection was begun cranially along the anterior surface of the sacrum.

During dissection, blood loss increased rapidly. At the onset of bleeding, the injury site could not be directly visualized; therefore, it was not possible to determine whether the main trunk or a branch of the right internal iliac vein had been injured or to identify the precise mechanism of injury. Manual compression was immediately applied. Because the systolic blood pressure decreased to 50–60 mmHg, massive transfusion was initiated promptly. The cumulative blood loss at this point was 1320 mL.

Compression alone did not provide adequate hemostasis. As tumor resection and dissection proceeded while hemostatic maneuvers were continued, a vein communicating with the right internal iliac vein was identified intraoperatively as entering the tumor. Because bleeding was profuse and the patient was hemodynamically unstable, rapid and reliable hemostasis was prioritized over vascular repair, and the main trunk of the right internal iliac vein near its junction with the right common iliac vein was ligated. Bleeding remained uncontrolled after venous ligation, and a branch arising from the right internal iliac artery was identified intraoperatively as entering the tumor. The specific name of this arterial branch could not be determined. This branch was ligated to permit tumor removal and obtain hemostasis; the main trunk of the right internal iliac artery was preserved. Because the tumor obstructed the operative view required for vascular control, life-saving hemostasis was prioritized over en bloc resection, and the tumor was removed in 2 pieces. The capsule was disrupted during this process, but no macroscopic tumor spillage into the operative field was observed.

After tumor removal, diffuse venous bleeding from the presacral venous plexus was encountered. Hemostasis was achieved using electrocautery and coverage with an oxidized cellulose hemostatic agent.

The operative time was 3 h 21 min, and the final intraoperative blood loss was 4850 mL. A total of 10 units of RBC-LR and 6 units of FFP-LR were transfused intraoperatively.

### Pathological findings

Macroscopically, the resected tumor consisted of 2 fragments measuring 85 × 75 × 25 mm and 60 × 35 × 20 mm. The cut surface was smooth, solid, elastic, and dark reddish, with admixed yellow fatty areas (**Fig. 4**). Hematoxylin and eosin staining showed a well-circumscribed lesion with a focal pseudocapsule-like structure at the periphery. Mature adipose tissue was admixed with hematopoietic cells, and the cellularity of the hematopoietic component was approximately 50% (**Fig. 5**). The hematopoietic cells showed no cytological atypia, and no malignant features were identified. Immunohistochemical staining for myeloperoxidase, glycophorin C, and CD61 highlighted myeloid, erythroid, and megakaryocytic cells, respectively, confirming trilineage hematopoiesis intimately admixed with mature adipose tissue (**Fig. 6**). These findings established the diagnosis of myelolipoma. Immunohistochemical staining for MDM2 and CDK4 and molecular testing for MDM2 amplification were not performed.

**Fig. 4:**
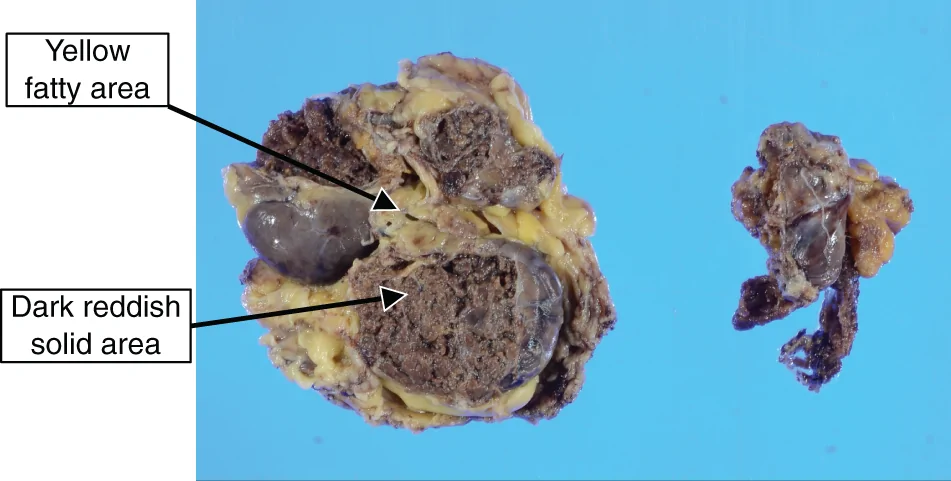
Macroscopic findings of the resected specimen. The cut surface shows a solid, dark reddish appearance admixed with yellow fatty areas.

**Fig. 5:**
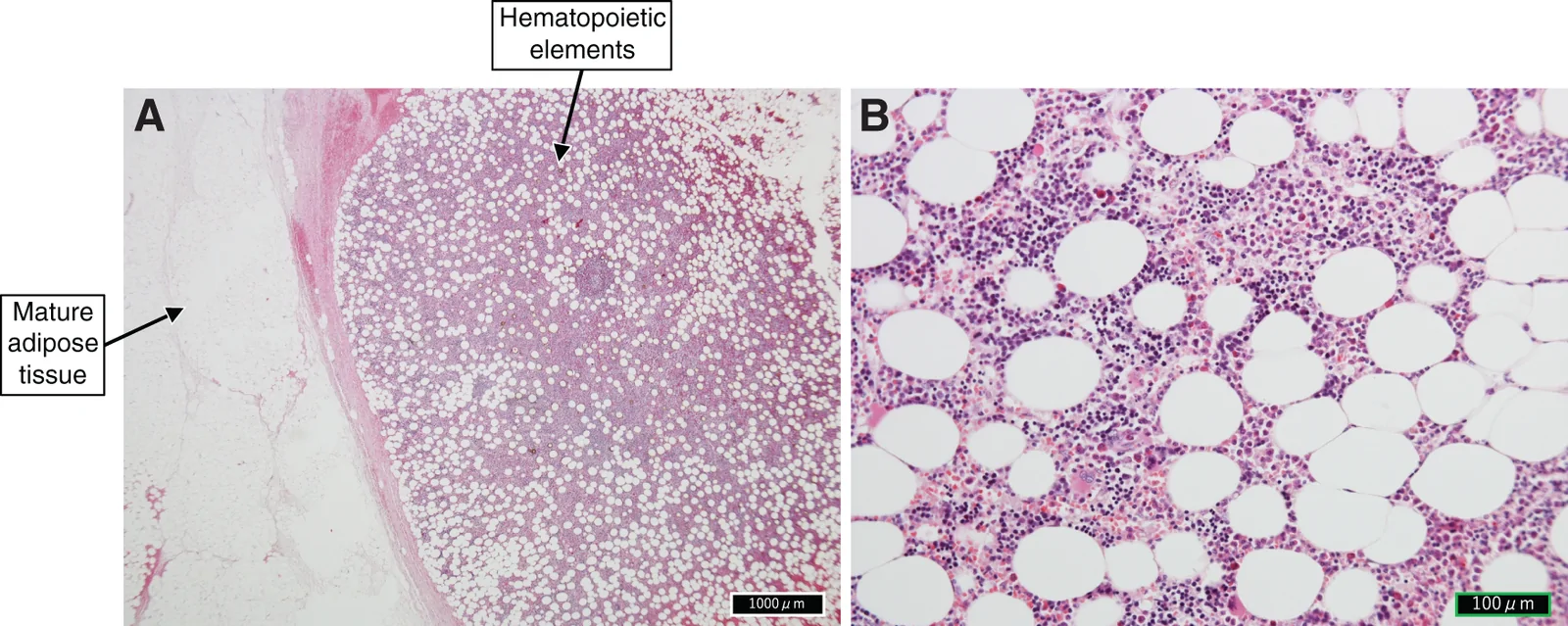
Histopathological findings with hematoxylin and eosin staining. (**A**) Low-power view showing an admixture of mature adipose tissue and hematopoietic tissue. (**B**) High-power view showing hematopoietic cells without cytological atypia. No malignant features are identified. Scale bars: (**A**) 1000 μm; (**B**) 100 μm.

**Fig. 6:**
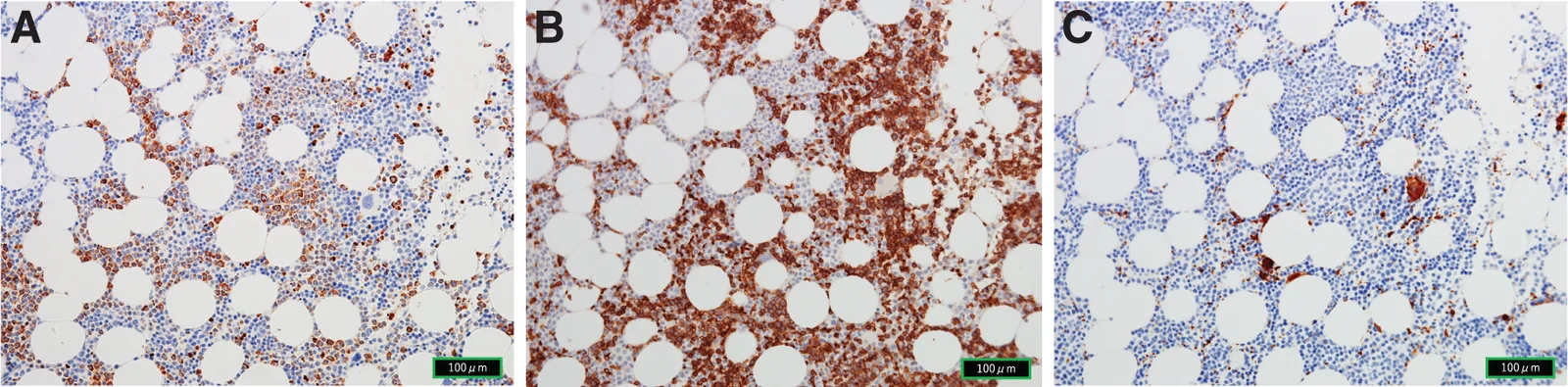
Immunohistochemical findings. (**A**) Myeloperoxidase staining highlights myeloid cells. (**B**) Glycophorin C staining highlights erythroid cells. (**C**) CD61 staining highlights megakaryocytes. These findings demonstrate trilineage hematopoiesis composed of erythroid, myeloid, and megakaryocytic lineages intimately admixed with mature adipose tissue. Scale bars: (**A**–**C**) 100 μm.

### Postoperative course

The hemoglobin level on the day after surgery was 10.3 g/dL, and no additional transfusion was required. The postoperative course was uneventful, and the patient was discharged on POD 16. After ligation of the main trunk of the right internal iliac vein near its junction with the right common iliac vein and an unnamed branch of the right internal iliac artery, no pelvic organ ischemia, buttock claudication, right lower-limb edema, deep vein thrombosis, pulmonary embolism, or urinary or defecatory dysfunction occurred. Contrast-enhanced CT performed approximately 1 year after surgery showed no residual or recurrent tumor and confirmed that the main trunk of the right internal iliac artery remained patent.

## DISCUSSION

The present case provides 2 clinically important lessons. First, presacral myelolipoma can closely mimic liposarcoma when it contains mixed fatty and solid components with diffusion restriction or FDG uptake. Second, dissection of a large presacral tumor can cause life-threatening hemorrhage from the internal iliac venous system and presacral venous plexus; preoperative imaging findings should therefore be translated into a specific hemostatic plan that includes blood product preparation and vascular control.

Myelolipoma is generally a benign lesion with an excellent prognosis, but extra-adrenal myelolipoma is uncommon.^1,2)^ In the autopsy series, adrenal myelolipoma has been found in approximately 0.08%–0.2% of cases and represents 6%–16% of adrenal incidentalomas.^1)^ Extra-adrenal lesions are much less common and have been estimated to account for approximately 15% of all myelolipomas.^4)^ A recent meta-analysis reported that fewer than 60 cases of presacral myelolipoma had been described in the English-language literature indexed in PubMed, with a female predominance and a mean age of 66.4 years.^4)^ The present patient was also an older woman with an incidentally detected presacral lesion, consistent with previously reported clinical features.

The main diagnostic challenge in this case was differentiation from liposarcoma. Myelolipoma typically appears as a well-circumscribed mass containing macroscopic fat and hematopoietic soft-tissue components on CT and MRI.^2,5)^ In contrast, well-differentiated liposarcoma may present as a fatty tumor with thick septa or nodular non-fatty components, and dedifferentiated liposarcoma may contain a more prominent solid component.^7,8)^ Although no short-term growth was observed in this case, the large tumor size, lobulated configuration, mixed fatty and solid components, and restricted diffusion prevented exclusion of liposarcoma. Continued observation was discussed as an option; however, the patient preferred definitive diagnosis and resection, and surgical treatment was selected.

FDG uptake also requires cautious interpretation. FDG-PET is widely used to help distinguish benign from malignant lesions, including adrenal masses.^9)^ However, adrenal myelolipomas can show low-level uptake in their non-fatty components.^6)^ In this patient, mild uptake with an SUVmax of 3.93 increased concern for malignancy before surgery, but the final histology suggested that the uptake likely corresponded to hematopoietic tissue rather than malignant transformation. Thus, FDG uptake alone should not be regarded as definitive evidence of malignancy in a fat-containing presacral tumor.

The indication for preoperative biopsy of a presacral tumor remains controversial. Biopsy may be useful when the result would alter management, such as when neoadjuvant therapy is considered, and when the biopsy tract can be excised during definitive surgery.^10)^ However, biopsy carries risks of infection, bleeding, tumor seeding, and sampling error. In a comprehensive review of retrorectal tumors, biopsy was performed in 27% of patients and yielded an inaccurate diagnosis in 44% of biopsied cases.^11)^ In the present case, the patient strongly preferred resection for both diagnosis and treatment, and the treatment plan was therefore surgical; consequently, the feasibility of image-guided biopsy was not formally assessed. In retrospect, because the patient was asymptomatic and no short-term growth had occurred, there was scope for more explicit consideration of continued observation or the feasibility of biopsy.

Pathologically, the differential diagnosis includes extramedullary hematopoiesis and well-differentiated or dedifferentiated liposarcoma. Both myelolipoma and extramedullary hematopoiesis can contain trilineage hematopoietic cells; however, myelolipoma generally forms a well-circumscribed mass in which mature adipose tissue and hematopoietic tissue are intimately admixed.^3)^ In the present case, there was no anemia, hemolytic disorder, myeloproliferative neoplasm, other hematologic disease, or hepatosplenomegaly, and hematoxylin and eosin and immunohistochemical staining demonstrated the characteristic admixture of mature adipose tissue and trilineage hematopoietic cells. No cytological atypia or other malignant features were identified. These findings supported a diagnosis of myelolipoma. In morphologically atypical or diagnostically difficult lipomatous tumors, MDM2 amplification by fluorescence in situ hybridization is considered more reliable than MDM2 immunohistochemistry as an ancillary test.^12,13)^ MDM2 and CDK4 immunohistochemistry and molecular testing for MDM2 amplification were not performed in this case, which is a limitation of the pathological assessment.

Massive hemorrhage during resection of myelolipoma is not confined to presacral disease. Omdal et al. reported an epidural myelolipoma extending from T1 to T10 that was highly vascular; resection caused more than 2500 mL of blood loss and required transfusion of 3 units of packed red blood cells.^14)^ In a series of 11 extra-adrenal myelolipomas arising in the thorax, paravertebral mediastinum, ilium, humerus, and rib, Cao et al. reported a mean intraoperative blood loss of 781.8 ± 1143.3 mL (range, 50–4000 mL); the 2 patients with blood losses of 1500 and 4000 mL required intraoperative transfusion, but no major postoperative complications occurred.^15)^ Liu et al. reported total blood loss of 3500 mL during emergency excision of a spontaneously ruptured 22-cm adrenal myelolipoma.^16)^ Although the mechanisms differ among spontaneous rupture, a highly vascular spinal canal lesion, and deep pelvic vascular injury, these reports demonstrate that the benign nature of myelolipoma does not guarantee a low perioperative bleeding risk. Tumor size, location, relationships with adjacent organs and vessels, and intratumoral vascularity should therefore be assessed individually.

Massive hemorrhage during presacral surgery should be considered according to whether it originates from injury to the presacral venous plexus or vertebral veins, or from injury to the main trunk or branches of the internal iliac vein. The presacral venous plexus comprises thin-walled veins adherent to the sacral periosteum; after injury, the bleeding point may retract, causing rapid and difficult-to-control hemorrhage.^10,17)^ Lou et al. described 4 cases of injury to the presacral venous plexus or vertebral veins during rectal surgery, with blood loss ranging from 600 to 6000 mL.^18)^ In contrast, Kieser et al. reported avulsion at adhesions between the internal iliac vein and neuroforaminal venous branches during sacrectomy; one patient had 15 L of blood loss and cardiac arrest, and another died intraoperatively despite massive transfusion.^19)^ These reports demonstrate that even within the presacral region, the hemostatic technique required depends on the bleeding source and mechanism of injury.

When presacral venous plexus bleeding occurs, direct pressure on the bleeding site, suction to maintain visualization, and simultaneous hemodynamic stabilization in collaboration with the anesthesiology team are fundamental.^18,20)^ Because direct suturing or indiscriminate coagulation of fragile venous walls may enlarge the injury, uncontrolled bleeding may require stepwise use of pelvic packing, circular suture ligation incorporating intact presacral fascia around the bleeding point, tacking, electrocoagulation through a muscle fragment, or topical hemostatic agents, according to the bleeding site and hemodynamic status.^20,21)^ If hemodynamic collapse develops with progressive hypothermia, acidosis, and coagulopathy, life-saving pelvic packing should be instituted promptly.^20)^ In the present case, diffuse bleeding from the presacral venous plexus after tumor removal was controlled with electrocautery and oxidized cellulose; however, readiness to proceed promptly to packing if these measures failed would also be important.

After surgery, retrospective review of the preoperative contrast-enhanced CT revealed a venous branch arising from the right internal iliac vein and coursing toward the tumor (**Fig. 1C**). This radiologic finding was not assumed to identify the precise vessel injured intraoperatively. The branch had not been recognized as a bleeding risk preoperatively, and 3D CT vascular reconstruction had not been performed; therefore, massive hemorrhage was not specifically anticipated. Preoperative recognition could nevertheless have prompted early identification and proximal control of the right common and internal iliac vessels, preparation of vascular repair instruments, and vascular surgical support. Planned control of the internal iliac vein and its major tributaries has been reported in high sacrectomy.^22)^ Intraoperatively, the main trunk of the right internal iliac vein near its junction with the right common iliac vein was identified and ligated for hemorrhage control. An arterial branch entering the tumor was also identified and ligated, although its specific name could not be determined; the main trunk of the right internal iliac artery was preserved. Only a type and screen was performed preoperatively; crossmatched RBC-LR was not reserved, and a massive transfusion system including FFP-LR was not prepared. For a large presacral tumor abutting pelvic vessels or containing a vessel coursing toward the tumor, preoperative planning should include thin-slice contrast-enhanced CT with a venous phase and multiplanar reconstruction, crossmatched RBC-LR and ABO-compatible FFP-LR, adequate large-bore venous access, arterial pressure monitoring, rapid transfusion and warming devices, vascular clamps and repair instruments, topical hemostatic agents, and availability of vascular surgical support before tumor mobilization.

Unilateral internal iliac artery occlusion is often tolerated because of the rich collateral circulation of the pelvis^23)^; however, this evidence is not directly applicable to the present patient because the arterial main trunk was preserved. Planned control of the internal iliac vein and its major tributaries has been described during pelvic resection.^22)^ Nevertheless, pelvic organ ischemia, buttock claudication, lower-limb edema, and deep vein thrombosis can occur after major pelvic vascular ligation; therefore, ligation cannot be considered uniformly safe, and repair is preferable for venous injury when the hemodynamic condition and injury morphology permit.^24)^ In the present case, none of these symptomatic complications occurred after ligation of the main trunk of the right internal iliac vein and an unnamed arterial branch. Follow-up contrast-enhanced CT at approximately 1 year confirmed patency of the main trunk of the right internal iliac artery. Postoperative monitoring for ischemia and venous congestion nevertheless remains necessary.

Complete resection of myelolipoma is usually curative, and recurrence after complete resection is rare.^4,25)^ In the present case, hemostasis was prioritized and the tumor was removed in 2 pieces. The capsule was disrupted, but no macroscopic tumor spillage into the operative field was observed. Because the specimen was fragmented, reliable histological assessment of the entire circumferential margin was not possible. Macroscopic completeness could not be confirmed retrospectively from the available operative documentation. Contrast-enhanced CT at approximately 1 year showed no residual or recurrent tumor; nevertheless, continued imaging follow-up is warranted.

## CONCLUSIONS

Presacral myelolipoma is a rare benign tumor that can be difficult to distinguish from liposarcoma when it contains mixed fatty and solid components with diffusion restriction or FDG uptake. Myelolipoma should be included in the differential diagnosis of a fat-containing presacral tumor, and surgical resection can provide both definitive diagnosis and treatment when malignancy cannot be excluded. Massive hemorrhage during myelolipoma resection has also been reported at other sites; therefore, bleeding risk should not be underestimated solely because the lesion is benign. In particular, resection of a large presacral tumor can cause fatal hemorrhage from both the internal iliac venous system and the presacral venous plexus. Detailed preoperative assessment of pelvic vessels, including branches coursing toward the tumor, adequate blood product preparation, early proximal vascular control, and a source-specific hemostatic strategy are essential.

## DECLARATIONS

### Availability of data and materials

Data sharing is not applicable to this article because no datasets were generated or analyzed during the current case report.
